# Soft Wireless Bioelectronics and Differential Electrodermal Activity for Home Sleep Monitoring

**DOI:** 10.3390/s21020354

**Published:** 2021-01-07

**Authors:** Hojoong Kim, Shinjae Kwon, Young-Tae Kwon, Woon-Hong Yeo

**Affiliations:** 1 George W. Woodruff School of Mechanical Engineering, Institute for Electronics and Nanotechnology, Georgia Institute of Technology, Atlanta, GA 30332, USA; hkim3023@gatech.edu (H.K.); skwon64@gatech.edu (S.K.); 2 Department for Metal Powder, Korea Institute of Materials Science, Changwon 51508, Korea; ykwon87@kims.re.kr; 3 Wallace H. Coulter Department of Biomedical Engineering and Parker H. Petit Institute for Bioengineering and Biosciences, Georgia Institute of Technology, Atlanta, GA 30332, USA; 4 Center for Human-Centric Interfaces and Engineering, Neural Engineering Center, Institute for Materials, and Institute for Robotics and Intelligent Machines, Georgia Institute of Technology, Atlanta, GA 30332, USA

**Keywords:** soft wireless sensor system, graphene electrode, galvanic skin response, sleep monitoring

## Abstract

Sleep is an essential element to human life, restoring the brain and body from accumulated fatigue from daily activities. Quantitative monitoring of daily sleep quality can provide critical feedback to evaluate human health and life patterns. However, the existing sleep assessment system using polysomnography is not available for a home sleep evaluation, while it requires multiple sensors, tabletop electronics, and sleep specialists. More importantly, the mandatory sleep in a designated lab facility disrupts a subject’s regular sleep pattern, which does not capture one’s everyday sleep behaviors. Recent studies report that galvanic skin response (GSR) measured on the skin can be one indicator to evaluate the sleep quality daily at home. However, the available GSR detection devices require rigid sensors wrapped on fingers along with separate electronic components for data acquisition, which can interrupt the normal sleep conditions. Here, we report a new class of materials, sensors, electronics, and packaging technologies to develop a wireless, soft electronic system that can measure GSR on the wrist. The single device platform that avoids wires, rigid sensors, and straps offers the maximum comfort to wear on the skin and minimize disruption of a subject’s sleep. A nanomaterial GSR sensor, printed on a soft elastomeric membrane, can have intimate contact with the skin to reduce motion artifact during sleep. A multi-layered flexible circuit mounted on top of the sensor provides a wireless, continuous, real-time recording of GSR to classify sleep stages, validated by the direct comparison with the standard method that measures other physiological signals. Collectively, the soft bioelectronic system shows great potential to be working as a portable, at-home sensor system for assessing sleep quality before a hospital visit.

## 1. Introduction

Sleep directly affects promoting human health and quality of life, which recuperates the brain and body from accrued daily fatigue [1,2]. Poor sleep quality can induce negative emotional response [3], increase daily stress [4], and degrade brain efficiency and memory preservation capability [5,6]. Continual sleep deficiency could reduce the immune system and escalate susceptibility to chronic infectious and neuropsychiatric diseases [7,8]. There are various ambulatory applications to analyze sleep quality and diagnose sleep disorders such as insomnia and sleep apnea [9,10,11]. Traditionally, polysomnography (PSG), using many sensors and electronics, is the gold-standard method for analyzing sleep quality [12,13]. The problem is that PSG requires a cumbersome, complicated setup of many electronic sensors, and the sleep study is carried out in a controlled laboratory with trained technicians. These requirements disrupt natural sleep patterns, and one-night sleep data is often not enough to represent normal sleep behavior.

Among non-invasive physiological signals, galvanic skin response (GSR) can be measured as a skin conductance controlled by the human body’s sympathetic nervous system [14,15]. Due to the relationship between the sensing mechanism of GSR and sleep regulated by the autonomic nervous system [16], GSR has been attracted as a simple, autonomic analytic solution for a sleep monitoring system. A recent study shows that GSR can determine sleep stages by monitoring the elevated signal frequency at deeper sleep stages [17,18,19]. A significantly lower GSR frequency was identified during the rapid eye movement phase [20,21]. Other papers analyzed GSR to classify the status between sleep and wake [22,23]. GSR signals have been measured on the fingers or palm area with multiple electrodes and data acquisition system [24], which allows the sensor location apart from the facial area that can disrupt natural sleep patterns, unlike the conventional PSG system. Although recent GSR devices are integrated with wearable electronics, they still rely on heavy and rigid sensors [25,26]. These devices require a tightly worn strap, band, or aggressive tapes to secure the sensor contact to the skin [27,28,29]. However, the mechanical mismatch of electronics and soft skin still causes motion artifacts and significant signal degradation during sleep.

Here, this paper introduces a fully-integrated, soft, wireless bioelectronic system that offers conformal lamination on the skin without the use of aggressive tapes and straps. The ultrathin, lightweight electronics allows high-quality, stable recording of GSR on the wrist during sleep with negligible motion artifact. A set of experimental studies captures the device’s mechanical flexibility and the quality of skin-electrode contact. The combination of signal processing and classification algorithms shows wearable bioelectronics’ capability to classify four sleep stages. Simultaneous recording and comparison of the GSR data with the conventional electroencephalogram (EEG) system validate the device performance.

## 2. Materials and Methods

### 2.1. Fabrication of a Soft Wearable Bioelectronic System

The fabrication of a soft electronic circuit followed the customized strategy reported in our prior works that used microfabrication and material transfer printing [30,31]. The overall process includes the following: polydimethylsiloxane/polyimide (PDMS/PI) was spin-coated on a 4-inch Si wafer. 1st layer of serpentine-shape pattered Cu was deposited by sputtering. Additional PI and 2nd Cu layer were laminated sequentially, followed by connecting the two Cu layers through via holes. After covering with the PI layer again, whole laminated PI layers were etched as serpentine-shape again to reduce tensile strain on the circuit when attaching on curved skin. The fabricated circuit was then transferred onto a soft silicone elastomer (1:2 mixture of Ecoflex 00-30 and Gels, Smooth-On, Macungie, PA, USA) by a water-soluble tape. Functional chip components and a rechargeable lithium-polymer battery (110 mAh, LP401230, Adafruit, New York, NY, USA) were integrated with the circuit, followed by encapsulating with the elastomer. For the fabrication of skin-contact flexible electrodes, we used a nanomaterial printing process based on aerosol jet printing [32,33,34]. A set of open mesh-shaped PI/graphene layers were printed on a polymethyl methacrylate (PMMA)-coated glass slide. After dissolving the PMMA in acetone, the electrode layers were transferred to a clinical-grade silicone tape (Kind Removal, 3M, St. Paul, MN, USA). The PI/graphene layers were linked to the circuit with a flexible connector. Details of the device fabrication steps appear in Supplementary Materials Note S1 and Figure S1.

### 2.2. Validation of Sleep Data with an EEG System

To validate the measured GSR in a sleep study, we made a simultaneous comparison of the fabricated bioelectronics with a conventional PSG system. For sleep quality recording, it is at least required to measure EEG, electrooculogram (EOG), and electromyogram (EMG) based on the contemporary American Academy of Sleep Medicine (AASM) guidelines [35]. Cloth gel-covered Ag/AgCl electrodes were placed on locations on a subject’s facial area for derivations of EEG (Fpz-M1), EOG (E1-E2), and chin EMG (Chin1-Chin2) as shown in Figure S2. Three nights of sleep were monitored by using both GSR and EEG devices at a subject’s home. The soft bioelectronics was powered by a rechargeable battery (capacity: 110 mAh) that could continuously record GSR data ~7 h during sleep (Figure S3). The GSR device was attached to the inner wrist of the subject’s non-dominant hand. For recording EEG, EOG, and EMG data, a customized circuit with nRF52 (Nordic Semiconductor, Trondheim, Norway) and ADS 1299 (Texas Instruments, Dallas, TX, USA) was used during a subject’s sleep. The study involved volunteers aged 18 and 40 and was conducted by following the approved IRB protocol (#H20211) at the Georgia Institute of Technology. Before the study, subjects agreed with the study procedures and provided signed consent forms.

### 2.3. GSR Data Acquisition

An nRF52832 microcontroller (Nordic Semiconductor, Trondheim, Norway) was used for data acquisition and storage. Raw data were stored directly onto the microcontroller’s flash memory. The sampling rate was set to 1 Hz to track changes during sleep periods of hours. The capacity of the available flash storage was 262 kB, which was available to store 12 h of data at a sampling rate of 1 Hz. The stored data was transmitted to an external device via a development kit (nRF52840 DK, Nordic Semiconductor, Trondheim, Norway) for further signal processing.

### 2.4. Quantification of Physiological Data

Raw GSR data was processed with the band-pass filter between 0.05 and 0.5 Hz to identify signal peaks. Averaged root mean square (RMS) was calculated from the filtered GSR to define a threshold level for sleep estimation and peak identification. During the sleep recording, the number of GSR peaks over the threshold level was counted within 30-s of an epoch. EEG and EOG data were band-pass filtered between 0.3 and 35 Hz, and chin EMG data were also bandpass-filtered between 10 and 100 Hz. The data were notch-filtered between 59.9 and 60.1 Hz to remove ambient power line 60 Hz noise. 30 s of sequential epochs throughout a dataset were scored based on visual observation of filtered signals and their multitaper spectrograms [36]. For statistics, the analysis of variance (ANOVA) was used to determine the significance between the sleep stages (α = 0.05).

## 3. Results and Discussion

### 3.1. Overview of a Soft Wearable Bioelectronic System for Home Sleep Monitoring

**Figure 1:**
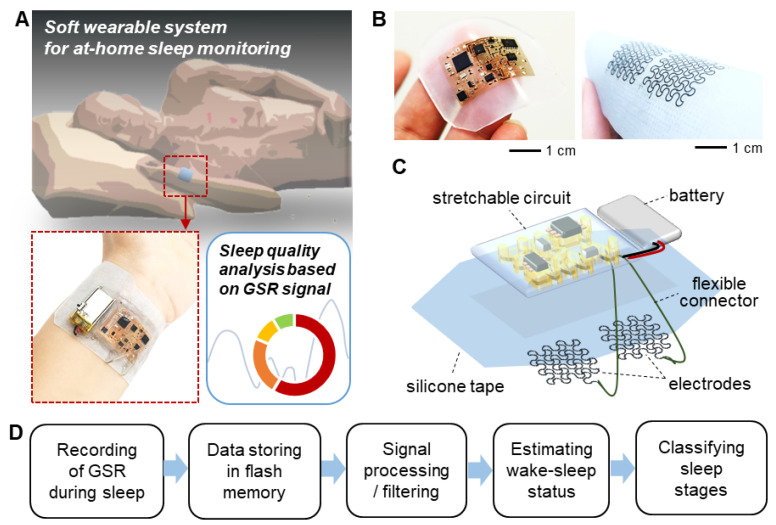
Overview of a soft wearable bioelectronic system for recording of electrodermal activity and home sleep monitoring. (**A**) Schematic illustration of a wearable patch on the wrist to assess sleep quality at home. (**B**) Photos showing a soft electronic system enclosed with an elastomer (left) and a pair of graphene electrodes on a silicone tape (right). (**C**) Illustration of the integrated bioelectronics, including a pair of electrodes, circuit, battery, and connector on a reusable silicone tape. (**D**) Flow chart that captures data recording and processing to quantify sleep stages at home.

Figure 1 captures the overview of a soft wearable system that can measure GSR signals to classify sleep stages at home. During sleep, the ultrathin, integrated device is mounted on the wrist (Figure 1A), which uses a biocompatible silicone tape. The fully-integrated, wireless device that is remarkably thin (<5 mm) and lightweight (<6.9 g with a rechargeable battery) offers comfortable wearability to the user during sleep while minimizing the disruption of the user’s regular sleep pattern. A flexible and breathable silicone tape enables the conformal adhesion of electrodes to the skin, reducing motion artifacts during the multi-hours period of measurement without skin irritation. Photos in Figure 1B capture the exceptionally small form factor and flexibility of the device. The integrated system has multiple components (Figure 1C), including a pair of printed graphene electrodes, flexible connectors, a rechargeable battery, and a stretchable circuit. The width and thickness of each electrode are 0.5 mm and 1 µm, respectively. The circuit detects GSR by measuring the potential variation in the Wheatstone bridge in the circuit. The Wheatstone bridge circuit can sensitively measure the potential difference between two parallel resistance paths, which are connected to the electrode pair. A digital potentiometer linked as a part of the Wheatstone bridge actively controls a signal baseline for increasing GSR variation amplitude (Figure S4). A silicone elastomer fully encapsulates the entire device except for the tape side. A flow chart in Figure 1D summarizes the overall procedure of estimating a sleep status and classifying sleep stages based on GSR data. Raw GSR signals are stored in the microcontroller chip’s flash memory in the circuit. The following steps work on signal processing and filtering to detect the transition between sleep and wake while evaluating four sleep stages.

### 3.2. Characterization of Mechanical and Electrical Properties of Printed Graphene Electrodes

**Figure 2:**
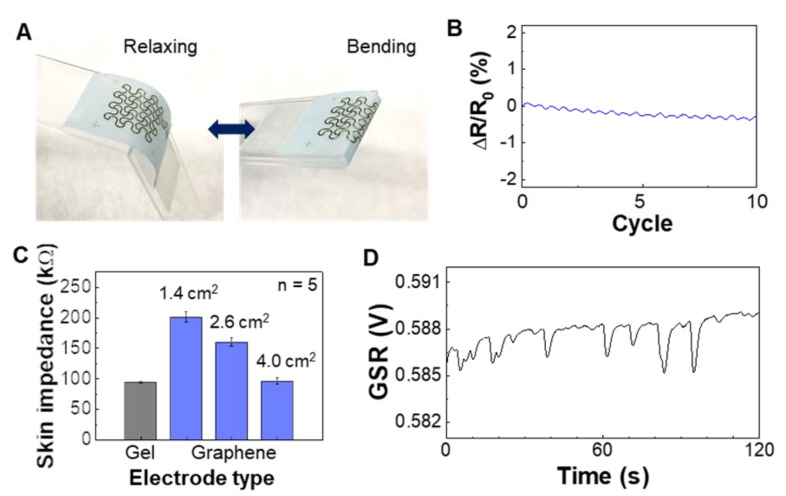
Characterization of mechanical and electrical properties of printed graphene electrodes. (**A**) Photos of a graphene electrode under cyclic bending. (**B**) Result of measured resistant change of the electrode in (**A**) with ten repetitive bending cycles up to 180 degrees. (**C**) Skin-electrode contact impedance on the wrist with different sizes of graphene electrodes and the comparison with a commercial gel electrode. Error bars indicate standard deviation from five trials (n = 5). (**D**) Measured GSR data from the wrist using the biggest graphene electrode (area = 4 cm^2^) in (**C**).

A set of experimental studies reveals the mechanical flexibility and quality of skin contact of printed graphene electrodes. We fabricated stretchable graphene electrodes via an aerosol jet printing [33,34]. Our prior work [32] shows the biocompatibility of graphene for the direct mounting of the material on human skin. Figure 2A shows a cyclic bending test (180 degrees with 2 mm in the radius of curvature) of the fabricated electrode that makes direct contact with the skin during GSR recording. The measured change of electrical resistance during cyclic bending shows negligible change below 0.5% (Figure 2B), indicating the electrode’s mechanical reliability. The bending induced strain on the wrist is 10–20% [37], and our prior work [32] shows the graphene’s stable performance up to 60% of tensile stress. Details of the experimental setup and measurement process appear in Figure S5, where we used a motorized force gauge and digital multimeter to detect the resistance change. A high-quality, accurate detection of GSR requires an intimate contact of the electrode with the skin, which is measured by the skin-electrode contact impedance (Figure 2C). We found out that an electrode covering 4 cm^2^ shows a comparable value with a conventional gel-based electrode by testing different electrodes’ sizes. As expected, the bigger electrode shows a lower impedance due to the increased skin-contact area. Figure 2D shows the measured GSR data on a subject’s wrist with the optimal size electrode from Figure 2C. The patch showed reliable adhesion over 6 h of wearing on the wrist and didn’t remain adhesive residues on the skin after sleep monitoring. The peeling energy of the silicone tape on the arm skin was 5.52 mJ, which is comparable to a conventional medical patch (Tegaderm, 3M, St. Paul, MN, USA), 9.16 mJ [38].

### 3.3. Detection of Wake-Sleep Transition Based on GSR Signals and Validation with EEG Signals

**Figure 3:**
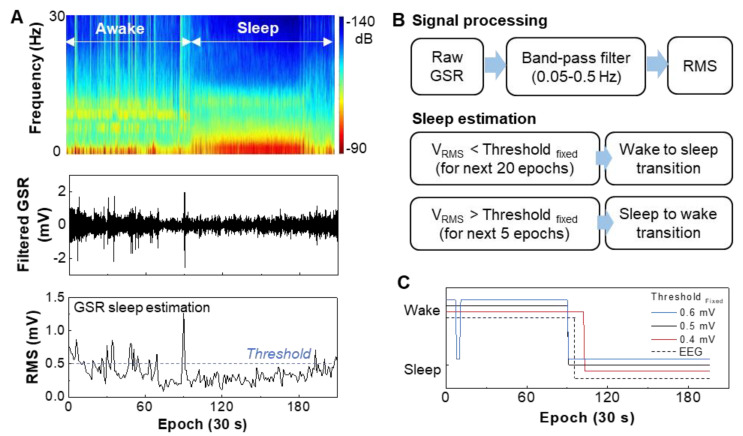
Determination of the wake-sleep transition based on GSR signals and validation with EEG signals. (**A**) EEG spectrogram measured on the forehead that differentiates the transition between awake and sleep (top), band-pass filtered GSR data measured on the wrist (middle), and RMS data from the filtered GSR for determination of sleeping (bottom). (**B**) Signal processing method of GSR data and sleep estimation process based on the RMS threshold. (**C**) Optimization of the threshold values to determine the sleeping transition in comparison with the EEG data.

This study focused on validating GSR recording device performance during sleep and correlation with EEG data measured simultaneously. While measuring GSR data on the wrist during sleep, a subject was wearing an EEG recording system and EOG and EMG electrodes to only extract sleep signals in EEG. Figure 3A shows the measured EEG and GSR data during sleep. The top graph shows the brain signals that clearly show the difference between awake and sleep states. GSR data measured from the wrist were going through the band-pass filtering, which shows a more fluctuating signal feature during wake than the stable data during sleep (middle graph, Figure 3A). For automatic detection of sleep/wake states, the filtered GSR data is converted to RMS values, and an averaged RMS is used as a threshold (bottom graph, Figure 3A). The calculated threshold is 0.51 ± 0.08 mV at n = 3 trials. Two flow charts in Figure 3B describe the details of the GSR signal processing steps and sleep state detection via the RMS threshold. When the RMS amplitude is below the threshold for a certain period, it is considered the wake to sleep transition. On the other hand, sleep to wake transition is defined when the RMS amplitude increases above the threshold. Long latency of 20 epochs is utilized to determine the onset of the wake to sleep transition compared to the sleep to wake (five epochs). This work’s sleep/wake evaluation method follows a prior report that analyzed sleep periods based on GSR [22]. Figure 3C summarizes the comparison between GSR data with varying thresholds and EEG-based sleep-wake detection. The threshold between 0.4 and 0.5 mV shows the best matching with the EEG data.

### 3.4. Determination of Four Sleep Stages Based on GSR Data

**Figure 4:**
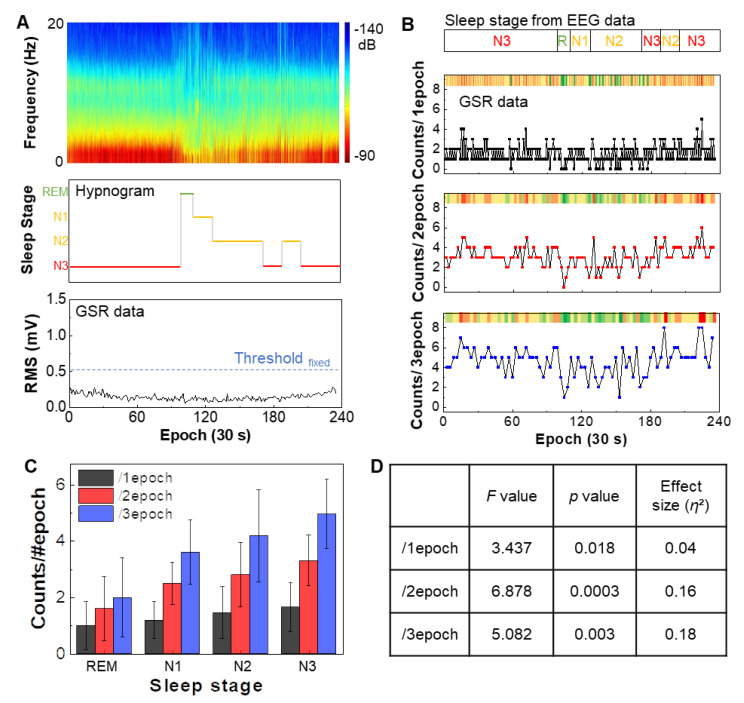
Determination of four sleep stages based on GSR data. (**A**) Raw EEG spectrogram measured during sleep (top), hypnogram derived from the EEG data showing four sleep stages, including REM, N1, N2, and N3 (middle), and measured GSR data during sleep (bottom). The RMS values from GSR data show a continuous low tendency below the fixed threshold, verifying the entire epochs are in sleep status. (**B**) Comparison of classified sleep stages from EEG data with GSR data in three epochs. (**C**) Summarized correlation between sleep stages and GSR counts from three epochs in (**B**). Error bars: standard deviation from three trials. (**D**) Summary of statistical analysis using ANOVA, showing the data significance in (**C**). High significance is identified in epoch 2 (*F* = 6.878, *p* = 0.0003, *η*^2^ = 0.16).

This study focused on the validation of sleep stage classification based on GSR data from the wrist. A subject had both wearable bioelectronics on the wrist and a set of EEG recording system during sleep. Even with multiple days of sleep, the device shows no adverse mechanical issues while enduring mechanical deformation on the skin. Figure 4A shows a comparison between EEG-based sleep classification and GSR recording. From the EEG spectrum, the hypnogram shows clear four stages of sleep, such as REM, N1, N2, and N3. The RMS signal from GSR shows continuous and low tendency below the fixed threshold level of 0.5 mV, verifying the entire epochs are in sleep status. In Figure 4B, the variation of the hypnogram’s sleep stages (top bar) is compared to a set of GSR signals (three sets of epochs in bottom graphs) during sleep. Averaged RMS calculated value from the filtered GSR is determined as a threshold for quantifying the GSR signals. The number of the filtered GSR peaks over the threshold level is counted per each epoch. Minimized noise signal without motion artifact is observed during sleep (Figure S6). Overall, counted peaks from GSR data are concentrated between 1 and 2, barely distinguishable as varying sleep stages. After increasing the window to 2 and 3 epochs, the GSR variation becomes noticeable. Relative color intensity (upper bar in each graph) denotes the GSR’s visualized evolution regarding the sleep stages. At the first N3 for 100 epochs, the counted peaks are relatively high (colors are yellow and red), which is decreased remarkably when the sleep transition to REM occurs (colors become green clearly). After passing through the moderate sleep levels, N1 and N2 (converted green to yellow gradually), the counted peaks rise again as the sleep stage develops deeper at 170 epochs (become yellow and red again). Figure 4C presents the correlation data between the sleep stages from EEG and the counted GSR peaks. Average counted numbers show the lowest value at the REM stage and increases as the sleep stage shifts to a deeper level. Overall this trend makes a great agreement with the prior work [17]. Figure 4D summarizes the analysis of variance (ANOVA) results to evaluate the significance among the sleep stages. Higher significance is observed in 2epoch (*F* = 6.878, *p* = 0.0003, *η*^2^ = 0.16) compared to other windows. Table 1 summarizes the comparison of wearable sleep monitoring devices measuring GSR data. Compared to the prior works, the soft bioelectronics presented in this work shows the novelty in the form factor, device flexibility, and sleep stage classification capability at home.

## 4. Conclusions

This work introduces a soft, wireless bioelectronic system along with a comprehensive study of soft materials, electronics, and packaging methods to fabricate at-home sleep monitor. The ultrathin, lightweight, single device platform allows high-quality, stable recording of GSR on the wrist during sleep. A set of experimental studies demonstrates the device’s mechanical flexibility for proper contact on the skin. The combination of signal processing and classification algorithm shows the wearable bioelectronics’ capability for the detection of sleep/wake transition and classification of four sleep stages. Simultaneous recording and comparison of the GSR data with the conventional EEG system validate the device performance. The fully-integrated, all-in-one soft electronics presented in this work have great potential as an at-home sleep monitoring and sleep quality screening system.

## Figures and Tables

**Table 1:** Comparison of wearable GSR-recording devices for a sleep study.

Ref.	Device Form Factor	Electrode Type	Data Recording	Device Location	Application	Recording Place	ANOVA
This work	Soft, thin, integrated	Dry (graphene)	Wireless	Wrist	Classification of sleep stages and Detection of sleep/wake states	Home	*F* = 6.9 *p* = 0.0003
[39]	Rigid and bulky	Dry (Ag)	Wireless	Wrist	GSR measurement during sleep	Home	-
[25]	Rigid and bulky	Gel (Ag/AgCl)	Wireless	Finger	GSR measurement during sleep	Lab	-
[23]	Rigid and bulky	Gel (Ag/AgCl)	Wired	Palm	Detection of sleep/wake states	Lab	-
[40]	Rigid and bulky	Dry (Ag)	Wireless	Wrist	Classification of sleep stages	Home	*F* = 55.8 *p* < 0.0001
[22]	Rigid and bulky	Gel (Ag/AgCl)	Wired	Finger	Estimation of sleep time	Lab	-
[17]	Rigid and bulky	Dry (Ag/AgCl)	Wireless	Wrist Palm	Classification of sleep stages	Home/Lab	*F* = 12.7 *p* < 0.00001

## Data Availability

The data presented in this study are available on request from the corresponding author.

## Supplementary Materials

The following are available online at https://www.mdpi.com/1424-8220/21/2/354/s1. Note S1. Fabrication of soft bioelectronics, Figure S1: illustration of the fabrication process of soft bioelectronics, Figure S2. System setup for the recording of EEG, EOG, and EMG signals during sleep, Figure S3. Battery lifetime, Figure S4. Schematic of circuit design and chip components arrangement, Figure S5. Experimental setup for mechanical reliability of graphene electrodes, Figure S6. Comparison of GSR signal to noise during sleep.
