# Supplementary material for: Soft Wireless Bioelectronics and Differential Electrodermal Activity for Home Sleep Monitoring
Source: Sensors (Basel). 2021 Jan 7;21(2):354. doi: 10.3390/s21020354 (PMC7825679; doi:10.3390/s21020354)
Supplement: Supplementary file 1 [file sensors-21-00354-s001.pdf]

Supplementary Materials

Soft Wireless Bioelectronics and Differential Electrodermal Activity for Home Sleep Monitoring

Hojoong Kim [1], Shinjae Kwon [1], Young-Tae Kwon [2], and Woon-Hong Yeo [1,3,4,*]

[1]George W. Woodruff School of Mechanical Engineering, Institute for Electronics and Nanotechnology, Georgia Institute of Technology, Atlanta, GA, 30332, USA; hkim3023@gatech.edu (H.K.); skwon64@gatech.edu (S.K.); whyeo@gatech.edu (W.H.Y.)
[2]Department for Metal Powder, Korea Institute of Materials Science, Changwon 51508, South Korea; ykwon87@kims.re.kr (Y-.T.K.)
[3]Wallace H. Coulter Department of Biomedical Engineering and Parker H. Petit Institute for Bioengineering and Biosciences, Georgia Institute of Technology, Atlanta, GA 30332, USA
[4]Center for Human-Centric Interfaces and Engineering, Neural Engineering Center, Institute for Materials, and Institute for Robotics and Intelligent Machines, Georgia Institute of Technology, Atlanta, GA 30332, USA

*Correspondence: whyeo@gatech.edu (W-.H.Y.); Tel.: +1-404-385-5710; Fax: +1-404-894-1658

Note S1. Fabrication of soft bioelectronics

Circuit fabrication

1. Spin-coat PDMS (4:1 base-curing-agent ratio) on a Si wafer at 4000 RPM for 30 s.
2. Oxygen plasma treatment on PDMS surface for 8 s.
3. Spin-coat 1st polyimide layer (PI, PI-2610, HD MicroSystems) at 2000 RPM for 60 s.
4. Soft bake at 100 °C for 5 min and hard bake at 250°C for 1 h.
5. Deposit 0.5 µm thickness of Cu by sputtering.
6. Spin-coat photoresist (PR, Microposit SC1813, MicroChem) at 3000 RPM for 30 s, and soft bake at 100°C for 5 min. Align with a photomask and expose UV light with intensity of 15 mJ/cm² for 12 s and develop with a developer (MF-319, MicroChem).
7. Etch Cu with Cu etchant (APS-100, Transene) and remove remaining PR with acetone, rinse with IPA and DI water.
8. Spin-coat 2nd PI layer (PI-2545, HD MicroSystems) at 2000 RPM for 60 s, and soft bake at 100°C for 5 min. Hard bake at 240 °C for 1 h in a vacuum oven.
9. Spin-coat PR (AZ P4620, Integrated Micro Materials) at 2000 RPM for 30 sec, and soft bake at 90°C for 4 min. Photolithography exposing UV light with intensity of 15 mJ/cm² for 100 s. Develop with a developer (AZ-400K, Integrated Micro Materials) diluted with DI water (AZ-400K:DI water = 1:4).
10. Etch for via hole with reactive ion etcher (RIE) at 250 W, 150 mTorr, and 20 sccm of oxygen for 15 min. Rinse with acetone, IPA, and DI water.
11. Deposit 2 µm thickness of 2nd Cu by sputtering.
12. Spin-coat PR (AZ P4620) at 1500 RPM for 30 s, and soft bake at 90°C for 4 min. Photolithography exposing UV light with intensity of 15 mJ/cm² for 120 s and develop.
13. Etch exposed Cu with Cu etchant. Remove PR with acetone, and rinse with IPA and DI water.
14. Spin-coat 3rd PI layer (PI-2610) at 3000 RPM for 60 s. Soft bake at 100°C for 5 min and hard bake at 240°C for 1 h in a vacuum oven.
15. Spin-coat PR (AZ P4620) at 900 RPM for 30 sec, and soft bakes at 90°C for 4 min. Photolithography exposing UV light with intensity of 15 mJ/cm² for 160 s and develop.
16. Etch exposed PI with RIE at 250 W, 150 mTorr, and 20 sccm of oxygen for 30 min. Remove remaining PR with acetone, and rinse with IPA and DI water.
17. Peel off the microfabricated circuit with a water-soluble tape (ASWT-2, Aquasol) from the PDMS/Si wafer and put it on the 1 mm thickness of silicone elastomer (1:2 mixture of Ecoflex 00-30 and Gels, Smooth-On). Washing the tape with DI water.
18. Mount microchip components with screen-print low-temperature solder paste (alloy of Sn/Bi/Ag (42%/57.6%/0.4%), ChipQuik Inc.). Bake solder at 170 °C for 2 min.
19. Download firmware and flash a device through program line connected to circuit with magnetic cubes.

Electrode printing

1. Spin-coat PMMA (950 PMMA, Kayaku Advanced Materials) on a slide glass at 1000 RPM for 30 s, bake at 200°C for 2 min.
2. Atomize a PI ink (PI-2610) dissolved in N-Methyl-2-Pyrrolidone (NMP) in pneumatic atomizer (Aerosol Jet 200, Optomec), deposit using a 300-µm-diameter nozzle, and then cure at 250°C.
3. Print graphene ink dissolved in the NMP with a 200-µm-diameter nozzle and cure at 200°C.
4. Dissolve the printed electrodes in an acetone bath overnight.
5. Peel off the printed graphene layer with a water-soluble tape (ASWT-2, Aquasol) from the PMMA/slide glass and put it on a silicone tape (Kind Removal, 3M). Washing the tape with DI water.

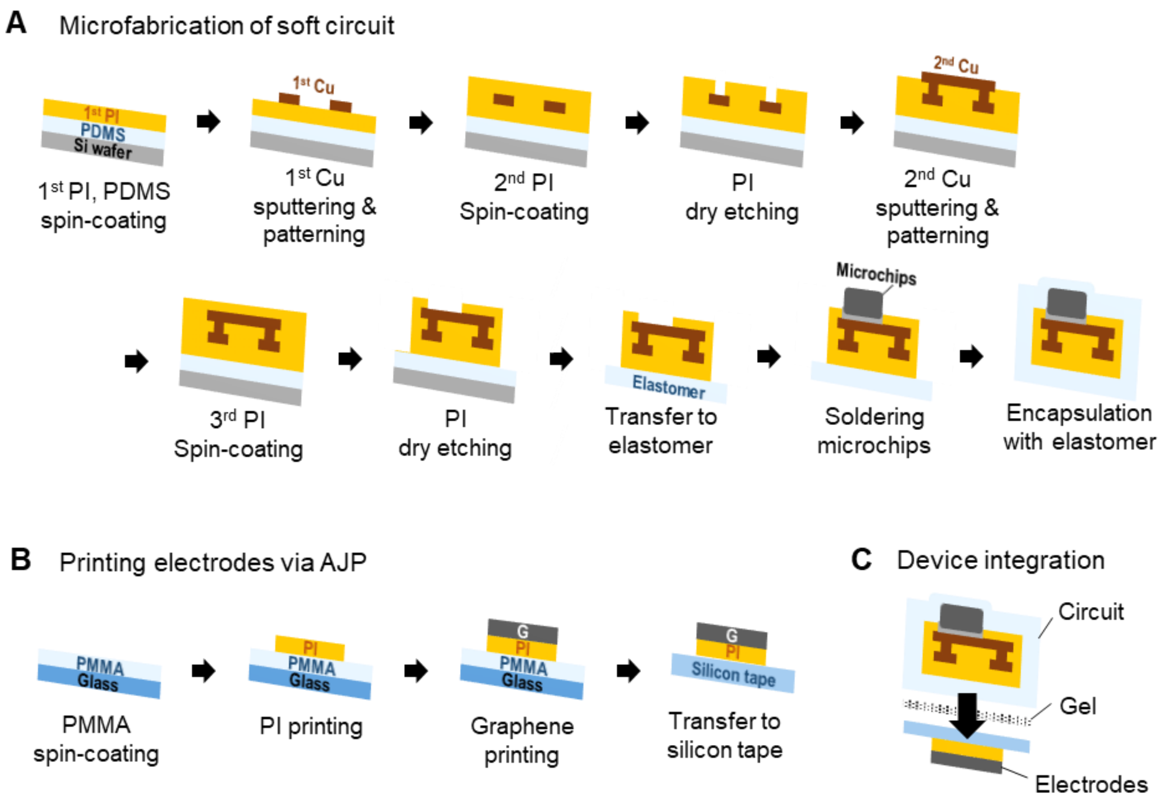

Figure S1. Illustration of the fabrication process of soft bioelectronics. (A) Stretchable serpentine-patterned circuit on a soft silicone elastomer. (B) Open-mesh graphene electrodes mounted on a soft silicone tape. (C) Device integration.

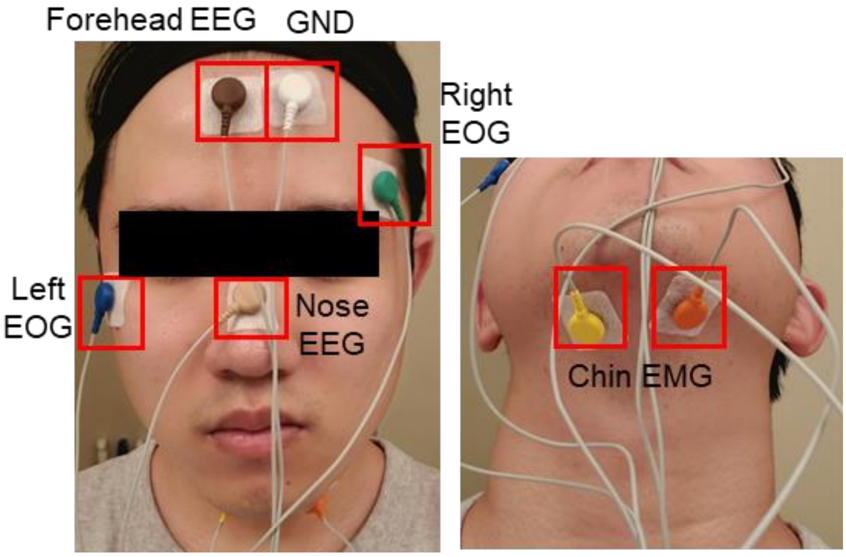

Figure S2. System setup for the recording of EEG, EOG, and EMG signals during sleep. Gel-covered Ag/AgCl electrodes (MVAP-2) were placed on locations on a subject's facial area for derivations of EEG (Fpz-M1), EOG (E1-E2), and chin EMG (Chin1-Chin2).

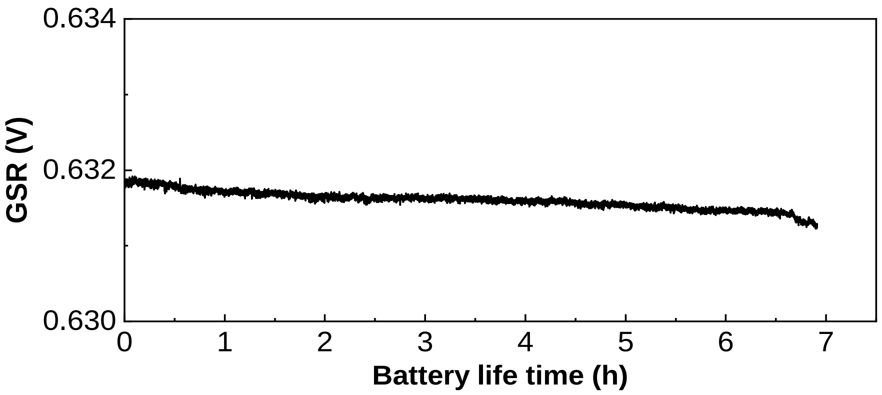

Figure S3. The battery lifetime of the sleep monitoring system with a fully charged 110 mAh LiPo battery. GSR signals can be recorded for 7 hours.

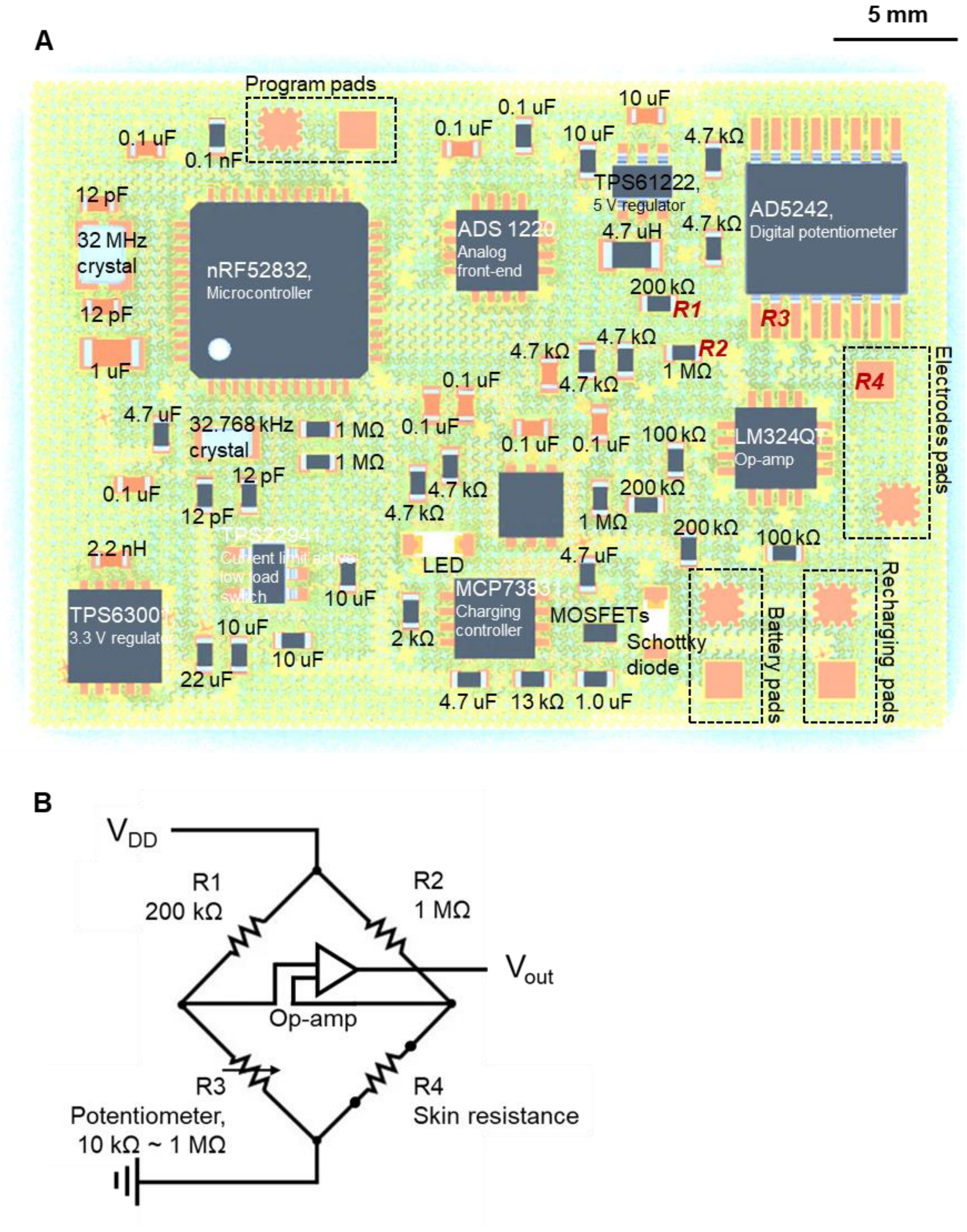

Figure S4. (A) The schematic of circuit design and chip components arrangement. R1-R4 represent the element of the Wheatstone bridge. (B) The circuit diagram of GSR ICs combined using the Wheatstone bridge, digital potentiometer, and op-amp for enhancing GSR sensitivity.

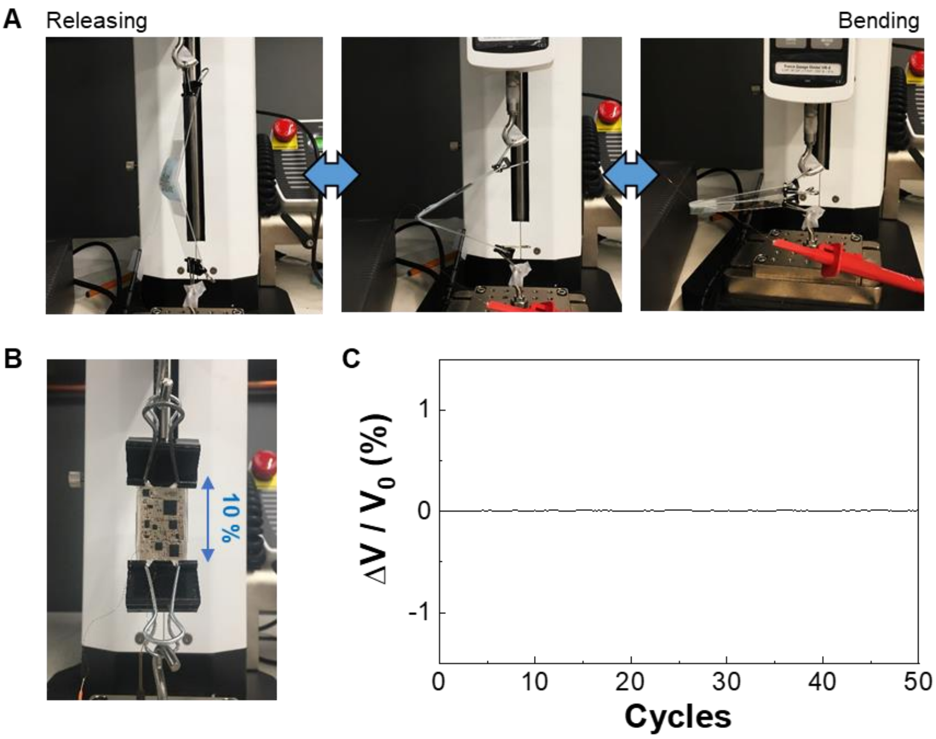

Figure S5. (A) Experimental setup for mechanical reliability of graphene electrodes. The electrodes incorporated on a silicone tape was mounted on a pair of slide glasses clamped on its edge to a stand. A programmable motorized force gauge (M5-5, Mark-10) applied constant bending cycles. Thin copper wires were connected at the edge of the graphene pattern for recording the change of electrical resistance during cycles. (B) Tensile strain test on the circuit to validate its stretchability. (C) Cyclic loading for 50 cycles with 10% strain showed the negligible signal variation of GSR baseline.

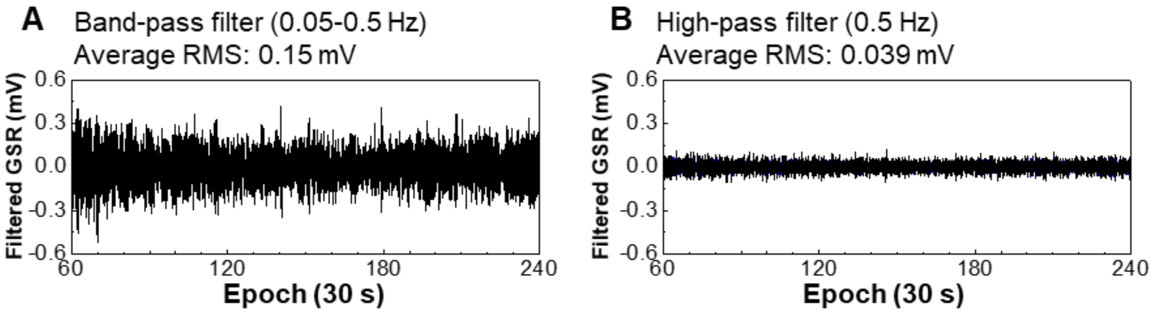

Figure S6. (A) The band-pass filtered (0.05-0.5 Hz) raw GSR data to quantifying the signal during sleep. (B) To calculate noise signal, we used the high-pass filter (0.5 Hz) to the raw GSR. Average RMS amplitudes of the signal and noise were calculated to 0.15 and 0.039 mV, respectively.
